# The EphB6 receptor is overexpressed in pediatric T cell acute lymphoblastic leukemia and increases its sensitivity to doxorubicin treatment

**DOI:** 10.1038/s41598-017-15200-3

**Published:** 2017-11-07

**Authors:** Amr El Zawily, Emily McEwen, Behzad Toosi, Frederick S. Vizeacoumar, Tanya Freywald, Franco J. Vizeacoumar, Andrew Freywald

**Affiliations:** 1 Department of Pathology and Laboratory Medicine, University of Saskatchewan, Royal University Hospital, 103 Hospital Drive, Saskatoon, SK S7N 0W8 Canada; 2 Department of Biochemistry, University of Saskatchewan, 107 Wiggins Road, Saskatoon, SK S7N 5E5 Canada; 3 Department of Oncology, University of Saskatchewan,107 Wiggins Road, Saskatoon, SK S7N 5E5 Canada; 4 Faculty of Science, Damanhour University, Damanhour, 22516 Egypt

## Abstract

While impressive improvements have been achieved in T-ALL therapy, current treatment approaches fail in approximately 25% of patients and these patients have limited treatment options. Another significant group of patients is being overtreated, which causes long-lasting side effects. Identification of molecules controlling drug resistance in T-ALL is crucial for treatment optimisation in both scenarios. We report here the EphB6 receptor is frequently overexpressed in T-ALL. Remarkably, our observations indicate that EphB6 acts in T-ALL cells to enhance sensitivity to a DNA-damaging drug, doxorubicin, as interruption of EphB6 activity interferes with the efficiency of doxorubicin-induced eradication of T-ALL cells in cell culture and in xenograft animals. This effect relies on the protection of Akt kinase signaling, while Akt inhibition combined with doxorubicin application produces synergistic effects on the elimination of EphB6-deficient T-ALL cells. These data imply that EphB6 suppresses T-ALL resistance by interfering with Akt activity. Our observations highlight a novel role for EphB6 in reducing drug resistance of T-ALL and suggest that doxorubicin treatment should produce better results if personalised based on EphB6 levels. If successfully verified in clinical studies, this approach should improve outcomes for T-ALL patients resistant to current therapies and for patients, who are being overtreated.

## Introduction

Pediatric T-cell Acute Lymphoblastic Leukemia (T-ALL) is an aggressive hematopoietic malignancy that originates from the transformation of immature thymocytes and is associated with the accumulation of multiple molecular abnormalities in the T cell lineage^1–3^. Genome-wide sequencing identified numerous mutations in T-ALL cells that affect molecules involved in cell cycle regulation, cytoplasmic signaling, and transcription regulation^3^. Among the most frequent genetic abnormalities found in T-ALL, are chromosomal deletions causing CDKN2A/2B inactivation and NOTCH1-activating mutations, each occurring in at least 50% of T-ALL cases^4,5^. NOTCH3 has been shown to be overexpressed in all examined T-ALL cases^6^, and expression levels of several transcription factors, including TAL1, LMO1, LMO2, BCL11B and HOX11 are also frequently altered in this malignancy^7–10^. These genetic alterations result in the development of T cells with several characteristics that drive malignancy, such as accelerated proliferation, enhanced cell survival, altered metabolism, and impaired differentiation^3^. The primary therapeutic strategy for T-ALL treatment is an intensive multiagent chemotherapy, which is effective in curing the disease in around 75% of patients^11,12^. Unfortunately, poor responsiveness to the initial therapy or cancer relapse are associated with an unfavorable prognosis in the remaining 25% of T-ALL patients. Therefore, there is an urgent need to identify molecules that determine drug resistance of T-ALL cells, as this knowledge should assist in improving existing treatment approaches.

The Eph group of receptor tyrosine kinases is presented by 16 Eph receptors, of which 14 are expressed in human cells^13–15^. Through their basal or ligand-induced action, these molecules control a vast variety of signaling events and cellular responses in both normal, and malignant cells^16^. Signaling activity of Eph receptors is modulated by their ligands, ephrins, where Eph receptors interact in a very promiscuous manner with ephrin-A (ephrin-A1 – ephrin-A6) or ephrin-B (ephrin-B1 – ephrinB3) types of ligands^17^. Based on their structural properties and ligand binding preferences, Eph receptors are divided into EphA or EphB subgroups, with EphA receptors binding mostly ephrin-A and EphB receptors interacting predominantly with ephrin-B molecules. In a classical model, ephrin binding induces Eph receptor dimerisation or oligomerization, leading to the phosphorylation on tyrosine residues, which further enhances catalytic activity of Eph receptors and supports their interaction with cytoplasmic signaling partners^13,16^.

Interestingly, two members of the Eph group, EphA10 and EphB6, are devoid of kinase activity because of several innate alterations in their kinase domains^15,18–20^. Accumulating evidence suggest that despite the lack of kinase activity, EphB6 undergoes tyrosine phosphorylation that can be provided by some catalytically active Eph receptors^21,22^ or Src family kinases^23^ and has important functions in T-lymphocytes^24–26^. Moreover, our previous work shows that along with the some other EphB receptors, EphB6 is also expressed in the majority of the analysed T-ALL cell lines and patient samples, where a collective action of EphB receptors protects T-ALL cells from Fas-induced apoptotic death^27^.

In our investigation discussed in this manuscript, we examined the role of EphB6 in T-ALL drug resistance. Our new findings reveal that EphB6 is the only member within the Eph group that is overexpressed in the majority of T-ALL cases. Remarkably, our observations also indicate that in contrast to the collective action of EphB receptors, EphB6 does not support survival of T-ALL cells, but rather increases their sensitivity both in cell culture and *in vivo* to a DNA-damaging therapeutic compound, doxorubicin, that is being commonly used for T-All treatment^11,28^. On a molecular level, this effect is associated with the decrease in the activating phosphorylation of a pro-survival molecule, the Akt kinase and with reduced phosphorylation of its downstream target, the p70 S6 kinase, indicating that EphB6 acts by suppressing Akt signaling in doxorubicin-treated T-ALL cells.

Taken together, our work identifies a previously undescribed function for the EphB6 receptor in controlling drug sensitivity of T-ALL cells and suggests that the efficiency of DNA-damaging therapeutic reagents, including doxorubicin, could be improved by applying them in a personalized manner to patients with different levels of EphB6 expression in T-ALL cells.

## Results

### The EphB6 receptor is overexpressed in the majority of T-ALL cases

**Figure 1:**
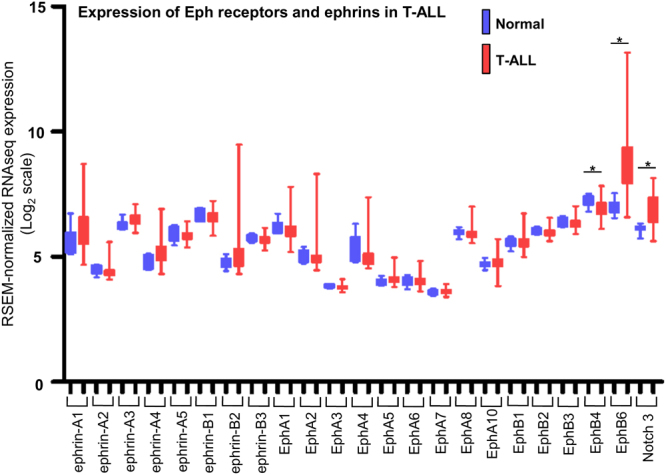
EPHB6 is overexpressed in T-ALL patient samples. Eph receptor and ephrin expression was analyzed in 117 pediatric T-ALL patient samples and 7 matching normal tissue controls using data from the European Bioinformatics Institute. Statistical significance was computed using the Mann-Whitney *U* test. Expression of the Notch3 receptor that is upregulated in T-ALL is shown as a reference.

Previously published observations, including the work of our team demonstrate that expression of the EphB6 receptor is maintained in most analysed T-ALL cell lines and some patient samples^27,29^. However, EphB6 expression, and expression of other Eph receptors and their ligands, ephrins, has never been systematically assessed in a larger collection of T-ALL cases in comparison to matching normal controls. To address this, we used the dataset from the European Bioinformatics Institute and analysed expression of ephrins and Eph receptors in 117 pediatric T-ALL patient samples, comparing it with matching normal tissue samples. The statistical significance of this analysis was verified using the Mann-Whitney *U* test. Excitingly, this approach revealed that EphB6 is the only molecule within the Eph/ephrin group, whose expression is significantly elevated in the majority of T-ALL cases (Fig. 1). Moreover, the quantification of our bioinformatics data also showed that EphB6 is overexpressed by at least two fold in ~76% of T-ALL cases, indicating that the EphB6 receptor may produce a prominent effect on the biological properties of T-ALL cells.

### EphB6 suppresses drug resistance of T-ALL cells

**Figure 2:**
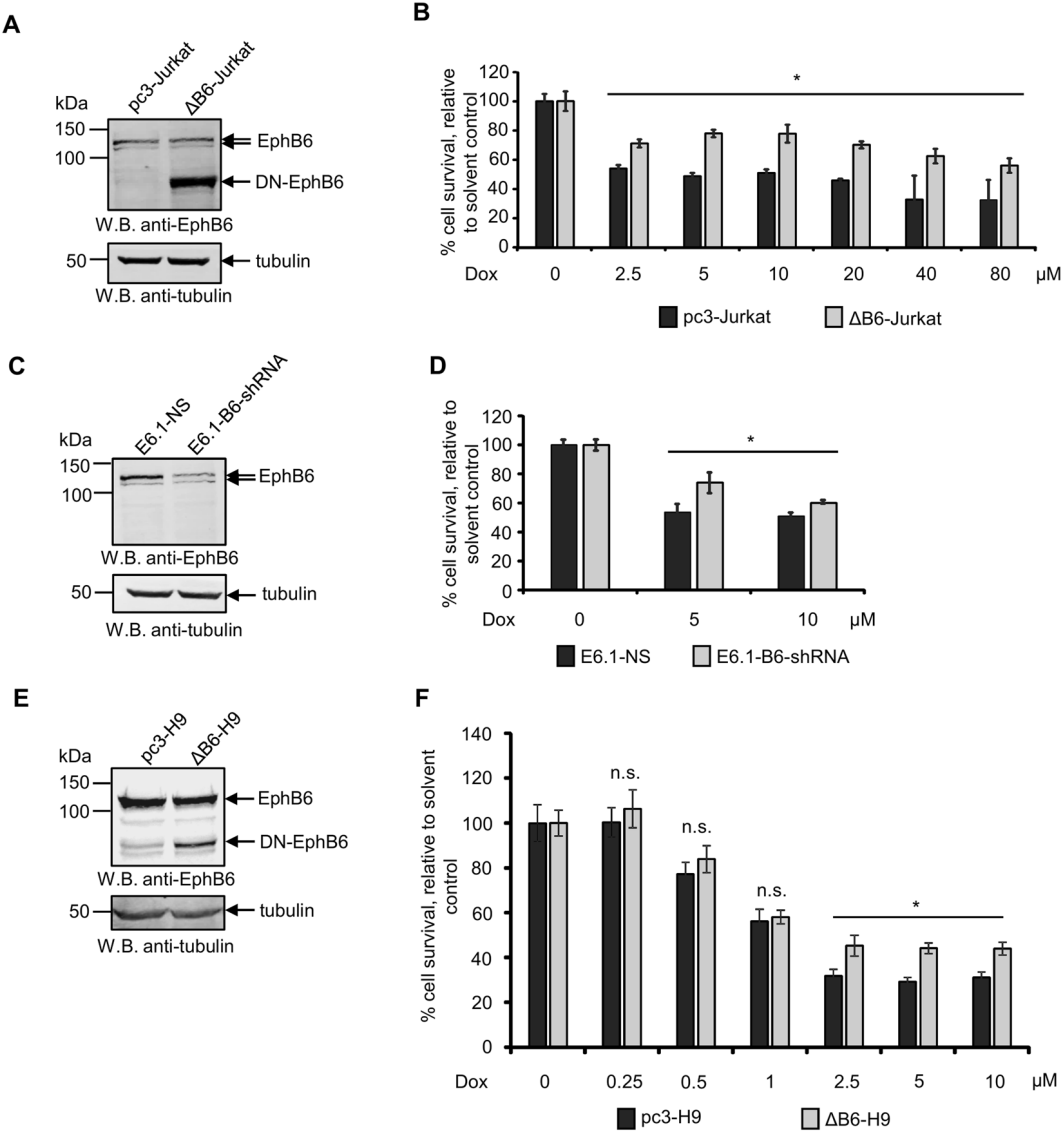
Suppression of EphB6 action supports survival of doxorubicin-treated cells. (**A**) Western blot analysis of EphB6 expression in Jurkat cells stably transfected with a cytoplasmic domain-deleted dominant-negative mutant (DN-EphB6) of EphB6 (ΔB6-Jurkat) or mock-transfected with the empty pcDNA3 expression vector (pc3-Jurkat). Western blotting with anti-tubulin was used as a loading control. (**B**) pc3-Jurkat and ΔB6-Jurkat cells (4 × 10^4^ cells per well in 96 well plates) were treated with the indicated doxorubicin (Dox) concentrations or a matching solvent control for 24 hours at 37 °C and 5% CO_2_. Cells were stained with resazurin and cell survival was quantitated using a SpectraMax M5 plate reader. The graph represents the analysis of five replicates and shows the percentage of survival of Dox-treated cells relative to matching solvent-treated cells. (**C**) E6.1 cells were transduced with EphB6-targeting shRNA (E6.1-B6-shRNA) or non-silencing shRNA (E6.1-NS). EphB6 expression was analysed by Western blotting with anti-EphB6. Western blotting with anti-tubulin was used as a loading control. (**D**) E6.1-NS and E6.1-B6-shRNA cells (4 × 10^4^ cells per well in 96 well plates) were treated with Dox or a matching solvent control for 24 hours. Cells were stained with resazurin and cell survival was measured using a SpectraMax M5 plate reader. The graph represents analysis of five replicates and shows the percentage of survival of Dox-treated cells relative to the survival of solvent-treated cells. (**E**) Human malignant lymphoblastic T cells, H9, were electroporated with the pcDNA3 expression vector encoding a DN-EphB6 mutant (ΔB6-H9) or mock-transfected with empty pcDNA3 as a control (pc3-H9), and cells were subjected to 30 days selection with 1 mg/ml of G418. Expression of DN-EphB6 was confirmed by Western blotting. (**F**) ΔB6-H9 and pc3-H9 cells were treated with the indicated concentrations of Dox or solvent control and cell survival was analysed by resazurin staining as in (**B**). All experiments were performed at least three times. To optimize presentation, anti-EphB6 and anti-tubulin Western blot images are shown at different brightness and contrast settings. Full-length unadjusted Western blot images for this figure are shown in Supplementary Fig. 1A–C. *P < 0.05, Student’s *t*-test. n.s., statistically not significant.

Our previous investigation showed that the collective action of EphB receptors inhibits Fas-induced apoptotic response in T-ALL cells^27^. Since we found that EphB6 is overexpressed in at least 76% of T-ALL patients, we suspected that it may also suppress apoptotic responses induced by chemotherapeutic drugs. To asses this possibility, we used in our experiments a previously described stable cell line originating from human T-ALL cells Jurkat, ΔB6-Jurkat, that had been transfected with a dominant-negative EphB6 mutant (DN-EphB6)^24^. This mutant maintains intact extracellular and transmembrane domains, but lacks the cytoplasmic portion and interferes with EphB6-mediated responses^24^. Jurkat cells mock-transfected with the empty pcDNA3 expression vector (pc3-Jurkat) were used as a specificity control (Fig. 2A). Contrary to our expectations, ΔB6-Jurkat proved to be significantly more resistant to the treatment with a DNA-damaging therapeutic compound, doxorubicin, which is being used in T-ALL therapy^11,28^, than control pc3-Jurkat cells (Fig. 2B). To confirm the specificity of this observation, we partially silenced EphB6 expression in T-ALL cells, E6.1, that have the same origin as Jurkat (Fig. 2C). Consistent with the effect of the DN-EphB6 mutant, EphB6 silencing also reduced sensitivity of T-ALL cells to doxorubicin (Fig. 2D). In these experiments, EphB6-targeting shRNA produced a relatively milder effect on increasing drug resistance, which was most probably due to the incomplete silencing of EphB6 expression (Fig. 2C). Taken together, these data indicated that EphB6 acts differently from other EphB receptors that collectively suppress apoptotic cell death in T-ALL cells and instead, enhances T-ALL sensitivity to doxorubicin treatment. This effect proved to be not cell line-specific, as expression of DN-EphB6 in human malignant lymphoblastic T cells, H9, also increased their resistance to doxorubicin (Fig. 2E and F).

### EphB6 action in increasing drug sensitivity of T-ALL cells is associated with enhanced apoptotic response and decreased Akt activation

**Figure 3:**
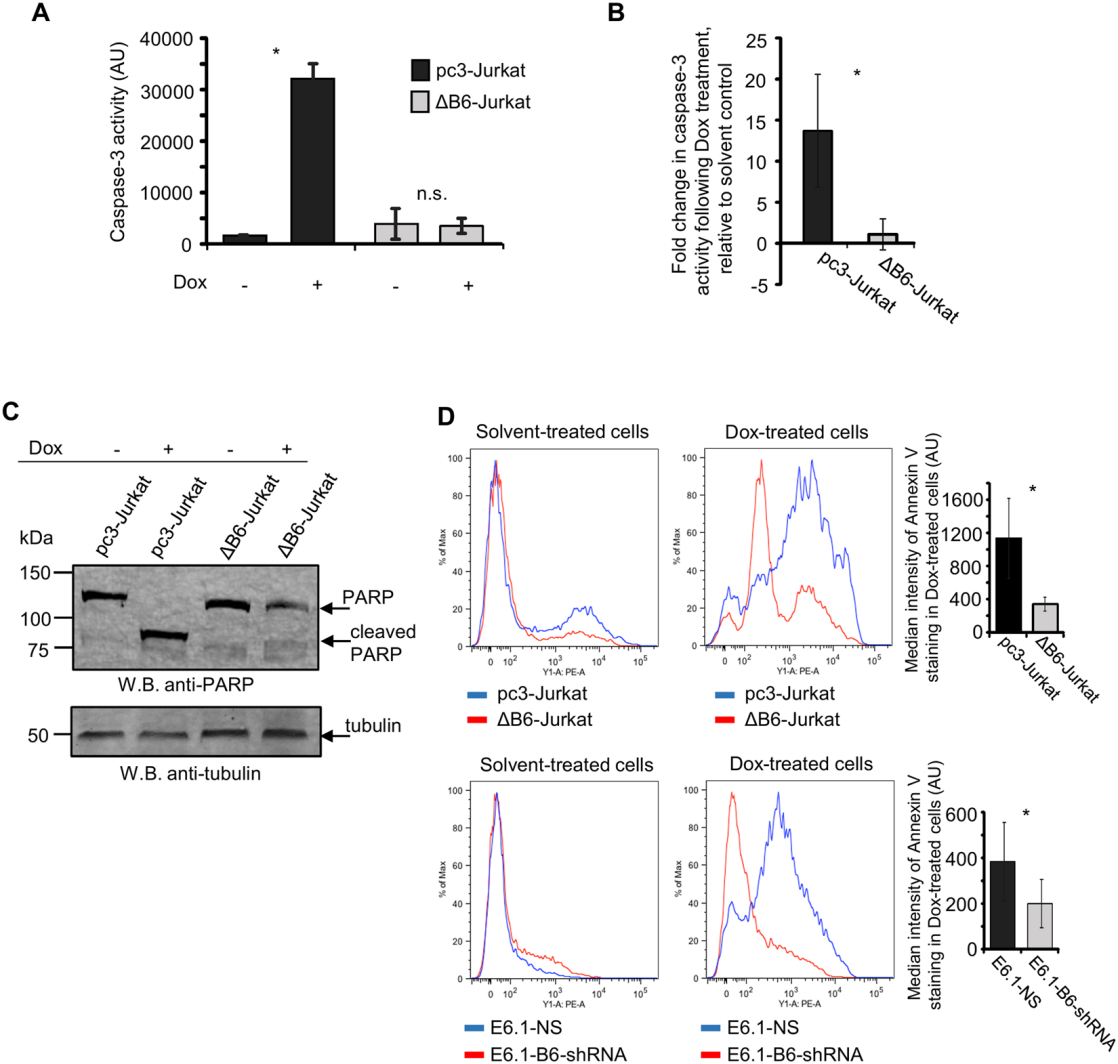
Disruption of EphB6 activity prevents induction of apoptosis in response to doxorubicin treatment. (**A**,**B**) ΔB6-Jurkat and pc3-Jurkat cells (1 × 10^6^ cells per well in 6 well plates) were treated in triplicates with 5 µM Dox or a matching solvent control in serum-free medium, for 14 hours. Caspase-3 activity was quantified using the EnzChek® CASPASE-3 Assay kit #1 (Invitrogen) according to the manufacturer’s instructions. (**A**) The graph shows caspase-3 activity in arbitrary units (AU), as measured at A_442_ using a SpectraMax M5 plate reader, and represents analysis of one of three independent experiments. (**B**) The graph shows fold-change in caspase-3 activity following Dox treatment, compared to a matching solvent control and represents analysis of three independent experiments. (**C**) ΔB6-Jurkat and pc3-Jurkat cells were treated with 5 µM Dox for 14 hours at 37 °C and 5% CO_2_. Cells were lysed and cell lysates were resolved by SDS PAGE. PARP cleavage was analysed by Western blotting with anti-PARP. Western blotting with anti-tubulin was used as a loading control. To optimize presentation, anti-PARP and anti-tubulin Western blot images are shown at different brightness and contrast settings. (**D**) The indicated cells were treated with 5 µM Dox, or a matching solvent control, for 24 hours. Cells were stained with PE-conjugated Annexin V for 15 minutes in the dark at room temperature and analyzed by flow cytometry. The graphs on the right show median intensities of Annexin V staining of Dox-treated cells after the subtraction of values corresponding to median staining intensities of matching solvent-treated controls. The graphs represent analyses of three independent experiments performed in duplicates. The analyses were done using the FlowJo software. All experiments were done at least three times. Full-length unadjusted Western blot images for this figure are shown in Supplementary Fig. 2A. *, *P* < 0.05, Student’s *t*-test. n.s., statistically not significant.

To determine if EphB6 increases drug sensitivity in T-ALL by supporting the induction of the apoptotic cell death, we monitored activation status of a key pro-apoptotic molecule, caspase-3 ^30^, in ΔB6-Jurkat and pc3-Jurkat cells using the EnzChek Caspase-3 assay. This approach revealed that inhibition of EphB6 activity strongly inhibits caspase-3 activation in doxorubicin-treated T-ALL cells (Fig. 3A and B). Consistent with this, expression of DN-EphB6 also blocked doxorubicin-induced cleavage of a caspase-3 substrate, poly (ADP-ribose) polymerase (PARP)^31^ (Fig. 3C). Moreover, our flow cytometry analysis of annexin V staining demonstrated that suppression of EphB6 action by the dominant negative mutant or shRNA-based silencing efficiently reduces doxorubicin-triggered apoptotic response (Fig. 3D).

**Figure 4:**
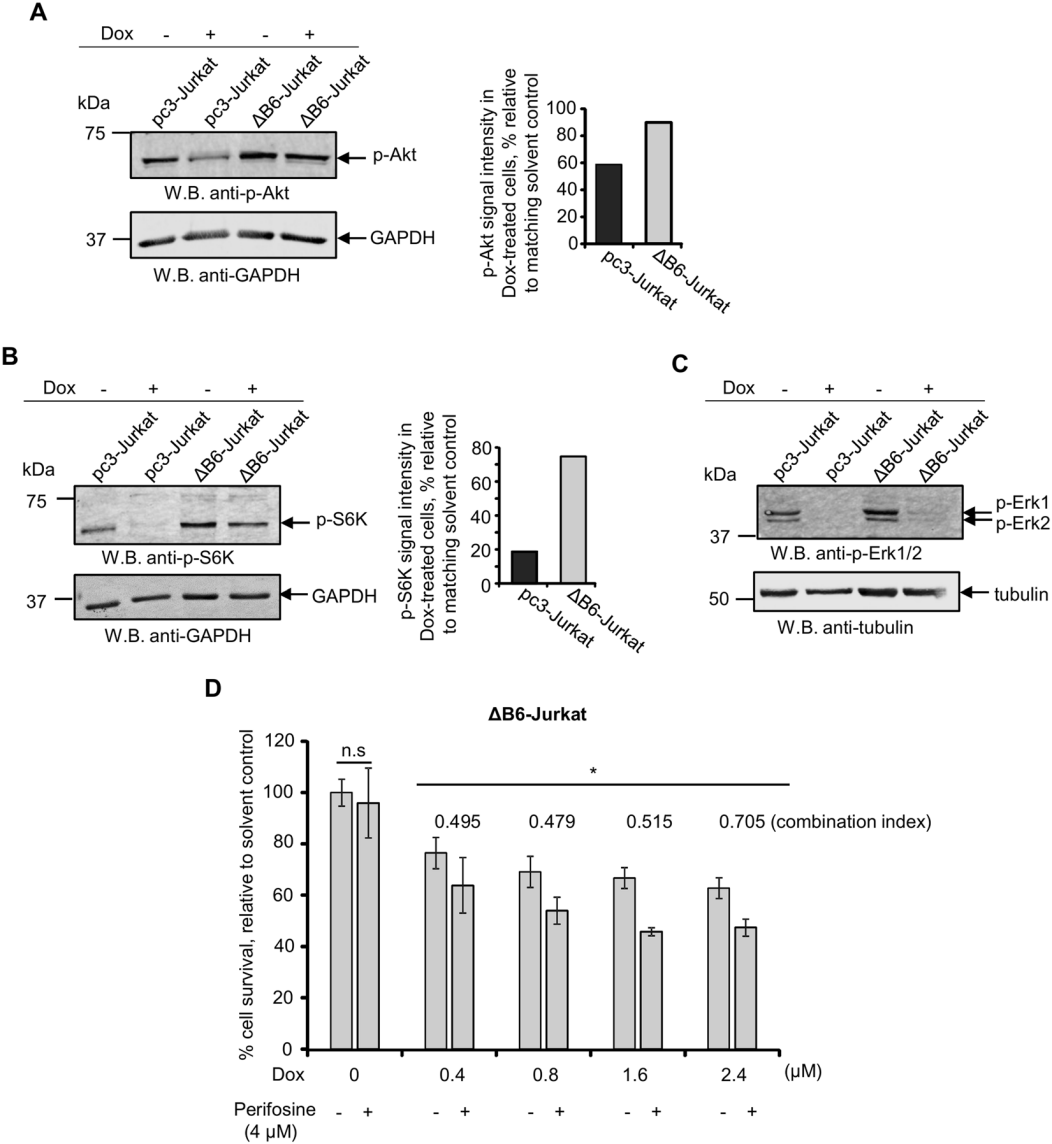
Expression of EphB6 dominant-negative mutant preserves Akt activation in doxorubicin-treated T-ALL cells. (**A**) ΔB6-Jurkat and pc3-Jurkat cells (4 × 10^6^ per well, in 6 well plates) were treated with 5 µM Dox in serum-free medium for 14 hours. Cell lysates were resolved by SDS PAGE and activating phosphorylation of the Akt kinase was examined by Western blotting with anti-phospho-Akt, recognising Akt phosphorylation at Ser473 (anti-p-Akt). Western blotting with anti-GAPDH was used as a loading control. Akt phosphorylation was quantified by densitometry using Carestream software and measurements were normalized to matching GAPDH controls. The graph represents a reduction in p-Akt signal intensity in Dox-treated cells as a percentage relative to the matching solvent controls. To optimize presentation, anti-p-Akt and anti-GAPDH images are shown at different brightness and contrast settings. (**B**) Cells were treated as in (**A**) and phosphorylation of the p70 S6 kinase was analysed by Western blotting with anti-phospho-S6K (anti-p-S6K). Phosphorylation of p70-S6K was quantified and presented as in (**A**). To optimize presentation, anti-p-S6K and anti-GAPDH images are shown at different brightness and contrast settings. (**C**) Cells were treated as in (**A**) and activating phosphorylation Erk kinases was analysed by Western blotting with anti-phospho-Erk (anti-p-Erk1/2). Western blotting with anti-tubulin used as a loading control. To optimize presentation, anti-p-Erk1/2 and anti-tubulin images are shown at different brightness and contrast settings. (**D**) ΔB6-Jurkat (4 × 10^4^ per well, in 96 well plates) were treated in triplicates with the indicated concentrations of Dox and with a suboptimal concentration of Perifosine individually, or in combination for 24 hours at 37 °C and 5% CO_2_. Cells were stained with resazurin and cell survival was monitored using a SpectraMax M5 plate reader. Data are shown as a percentage of cell survival in drug-treated populations relative to matching solvent controls. The synergistic effect of Dox/Perifosine combinations was calculated using CompuSyn software and is presented as combination indices for each drug combination. Indices below 0.9 indicate synergism. All experiments were done at least three times. Full-length unadjusted Western blot images for this figure are shown in Supplementary Fig. 3A–C. *, *P* < 0.05, Student’s *t*-test. n.s., statistically not significant.

Induction of the apoptotic cell death is negatively regulated through the activation of the Akt kinase and therefore, we assessed activating phosphorylation of this molecule on Ser473 residue by Western blotting with a phospho-specific antibody. In line with the pro-apoptotic action of EphB6, interruption of its signaling was associated with the lack of decrease in Akt phosphorylation in doxorubicin-treated cells (Fig. 4A). This was also accompanied by the maintained level of phosphorylation of a downstream target of the Akt-initiated signaling cascade, p70 S6 kinase^32^ (Fig. 4B). The EphB6 receptor appeared to affect Akt signaling in a selective manner, as no effect of DN-EphB6 on activating phosphorylation of Erk kinases that also support cell survival^33^ could be observed in doxorubicin-treated cells (Fig. 4C). Consistent with the model, where disruption of EphB6 signaling rescues T-ALL cells by protecting Akt activation, co-administration of doxorubicin and Akt inhibitor, perifosine, synergistically enhanced elimination of ΔB6-Jurkat cells (Fig. 4D).

Overall, these observations strongly suggest that EphB6 increases drug sensitivity by suppressing Akt signaling in doxorubicin-treated T-ALL cells, which ultimately enhances the apoptotic response.

### Interruption of EphB6 signaling increases drug resistance of T-ALL cells *in vivo*

**Figure 5:**
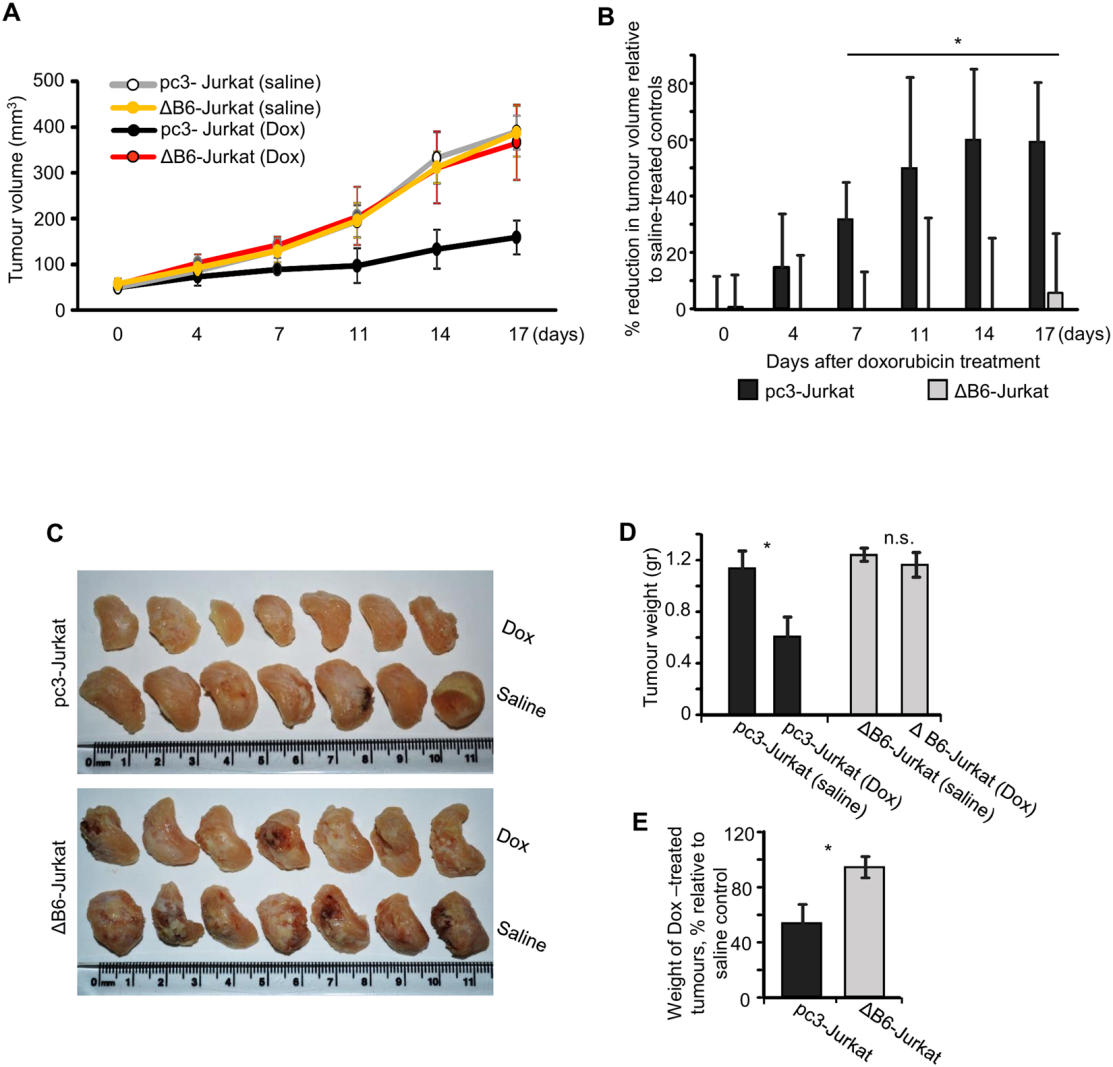
Inhibition of EphB6 action enhances drug-resistance of T-ALL cells *in vivo*. (**A**–**E**) ΔB6-Jurkat and pc3-Jurkat cells were resuspended in PBS and mixed 1:1 with Corning Matrigel Matrix. Cells (3 × 10^6^) were injected in a total volume of 100 µL subcutaneously into the right flank of NOD-SCID mice. As tumours reached a measurable size, mice were treated weekly with intravenous injections of Dox (2 mg/kg) or a matching volume of saline control (n = 7 per group). Tumour growth was monitored twice a week by measurements with digital callipers and tumour volume was calculated by the equation: A/2*B^2^, where A was long and B was short diameter of the tumour (**A**). The reduction in tumour growth in Dox-treated mice is presented as a percentage relative to matching saline-treated controls (**B**). In panels (A and B), day 0 indicates the day, when both treatment and measurements were initiated. Upon experiment termination, tumours were extracted, fixed in 10% formalin, photographed (**C**) and weighed. The average tumour weight for each experimental group is shown (**D**). The graph in panel (E) represents average tumour weights in Dox-treated mice as a percentage relative to matching saline-treated controls. Data are shown as means ± SD. One of two independent experiments is shown. *P < 0.05; Student’s *t*-test. n.s., statistically not significant.

The ultimate test for the drug sensitivity of cancer cells should be performed in an *in vivo* model. To achieve this, we followed previously described protocols^34,35^ and injected ΔB6-Jurkat and pc3-Jurkat cells pre-mixed with matrigel into the flanks of immunodificient NOD.Cg-Prkdc^scid^ Il2rg^tm1Wjl^/SzJ (NOD-SCID) mice. Resulting tumours were allowed to develop to a measurable size and treated with intravenous tail injections of doxorubicin. While expression of DN-EphB6 has not affected tumour growth (Fig. 5A), it dramatically decreased the effectiveness of doxorubicin treatment (Fig. 5B–E). These *in vivo* data matched our initial observations obtained in cultured cells and provided a strong support for a model, whereby EphB6 action suppresses T-ALL resistance to treatment with DNA-damaging reagents.

## Discussion

Acute lymphoblastic leukemia (ALL) is the most common pediatric malignancy and is the main cause of disease-related death in children. T-ALL is an aggressive ALL subtype that constitutes approximately 15% of pediatric ALL cases. During the last several decades, gradual fine-tuning of combination chemotherapeutic approaches dramatically improved T-ALL treatment and patient survival. Nevertheless, current treatment protocols fail in approximately 25% of T-ALL patients, and these patients cannot be cured^2,36^. Moreover, approximately 25% of children with T-ALL responding to chemotherapy are currently being overtreated, when unnecessarily aggressive therapy is being applied^12^. This over-aggressive treatment is often associated with severe long-lasting adverse side effects. All these deficiencies of current treatment protocols clearly indicate a strong need for more personalised therapeutic approaches and for less toxic targeted therapies. Identification and characterisation of molecular mechanisms that control responsiveness of T-ALL cells to chemotherapeutic reagents is crucial for treatment personalization, and for the development of effective targeted therapeutic strategies. In our work presented here, we found that a kinase-inactive member of the Eph group of receptor tyrosine kinases is the only Eph receptor that is significantly overexpressed in the majority of T-ALL cells. Since very high levels of EphB6 expression were previously observed in thymus and thymocytes^19,29^, it is likely that EphB6 overexpression does not directly contribute to T-ALL development, but rather represents a bystander effect of thymocyte transformation and reflects immature phenotypes of T-ALL cells. This matches our observations, showing that EphB6 does not accelerate propagation of T-ALL cells in a xenograft model and instead, reduces aggressiveness of T-ALL cells by making them more sensitive to the treatment with a DNA-damaging chemotherapeutic drug, doxorubicin. Obviously at this stage, we cannot completely exclude a possibility that EphB6 overexpression supports malignant transformation in the T cell lineage and this possibility is currently under an active investigation in our laboratory. Our previous work showed that EphB receptors collectively act through enhancing Akt activation to suppress Fas-induced apoptosis and support survival of T-ALL cells^27^. Interestingly, our current data reveals that EphB6 has a completely opposite function in T-ALL cells, since inhibition of its action through the expression of a dominant-negative mutant or by shRNA-based silencing inhibits doxorubicin-triggered apoptotic cell death and this effect is supported by the increased Akt activation. This is consistent with our earlier observations, showing that EphB6 interacts with at least some kinase-active EphB receptors^21,22^ and switches their pro-malignant action in cancer cells into anti-malignant responses^22^. Potential clinical relevance of our findings is further supported by *in vivo* experiments, confirming that EphB6 enhances doxorubicin-induced eradication of T-ALL cells not only in cell culture, but also in animal models. At this stage, our findings deliver an important message, suggesting that T-ALL treatment with DNA-damaging chemotherapeutic compounds could be efficiently personalised based on the level of EphB6 expression in T-ALL cells. Our observations suggest that patients with lower EphB6 levels could benefit from higher doses of doxorubicin, while lower doses could be sufficient for treating patients with high EphB6 expression. This approach could help to overcome resistance in some T-ALL cases, while also reducing undesirable side effects in patients, who are currently being overtreated. A careful matching of required doxorubicin doses to EphB6 levels in patients’ T-ALL cells would be required in future for developing personalised treatment protocols.

## Materials and Methods

### Antibodies and reagents

Anti-EphB6 was obtained from Santa Cruz (Dallas, TX, USA). Anti-phospho-Akt (Ser473), anti-phospho-S6K, anti-phospho-ERK1/2, and anti-GAPDH were purchased from Cell Signaling Technology (New England Biolabs Ltd., Whitby, ON, Canada). Anti-PARP was purchased from EMD Millipore (Billerica, Mass, USA). Anti-β-tubulin was obtained from Santa Cruz Biotechnology (Dallas, TX, USA). Bovine serum albumin (BSA) was purchased from BioShop Canada Inc. (Burlington, ON, Canada). Doxorubicin was purchased from Tocris Bioscience (Bristol, UK). Puromycin and polybrene were bought from Sigma-Aldrich (Oakville, ON, Canada). Resazurin was purchased from R&D Systems (Minneapolis, MN, USA). Perifosine was from Invivogen (San Diego, CA, USA). Tween-20 was from Fisher Scientific (Ottawa, ON, Canada).

### Western blot analysis

Cell lysates were prepared by washing cells once with ice-cold phosphate buffered saline (PBS) and lysing them in lysis buffer (0.2% NP-40, 5 mM EDTA, 20 mM Tris,150 mM NaCl). Halt Protease and Phosphatase Inhibitor Cocktail (Thermo Fisher Scientific) was added to the lysis buffer according to the manufacturer’s instructions and lysates were left for 30 minutes on ice. Cell debris was removed by centrifugation. Samples were resolved by SDS-PAGE, and transferred to a nitrocellulose membrane (Amersham, GE Healthcare Life Sciences, Baie d’Urfe, QC, Canada). Membranes were blocked in TBS (50 mM Tris Base, pH = 7.4, 150 mM NaCl) containing 0.1% Tween-20 and 7% BSA, or in PBS with 0.1% Tween-20 and 7% non-fat dry milk, and then incubated with a primary antibody overnight on a shaker at 4 °C. Membranes were washed three times with TBS or PBS and incubated for 1 hour with a fluorescently labeled secondary antibody in TBS or PBS containing 0.1% Tween-20 and 5% BSA or 5% non-fat dry milk. Following three additional washes with TBS or PBS, membranes were imaged with the LI-COR Odyssey imaging system (LI-COR Biotechnology, Guelph, ON, Canada). Fluorescence intensity was analysed by densitometry using the Carestream software (Carestream Molecular Imaging Software, New Haven, CT, USA). Figures were generated using PowerPoint software. Western Blot images were cropped using PowerPoint. Brightness and contrast of Western blot images were adjusted using the PowerPoint software to optimize image presentation.

### Cell culture

H9 and T-ALL cells E6.1 were purchased from ATCC (Manassas, VA, USA). Stable T-ALL cell lines, ΔB6-Jurkat, pc3-Jurkat and DN-EphB6 construct cloned in the pcDNA3 expression vector were described previously^24^ and were a kind gift from Dr. Chaim Roifman, Hospital for Sick Children, Toronto, Ontario. T-ALL cells were cultured in RPMI-1640 (HyClone, Logan, UT) medium supplemented with 10% FBS and 1% Pen-Strep (HyClone, Logan, UT) at 37 °C and 5% CO_2_.

### Lentiviral transduction

Lentiviral particles encoding EphB6-targeting shRNA (Sigma-Aldrich, St. Louis, MO, USA) or non-silencing shRNA (Sigma-Aldrich), were generated by co-transfecting HEK-293T cells with the pMDLg/pRRE (1.7 μg), pMD2G (1.11 μg), pRSV-Rev (1.7 μg) plasmids, and 4.17 μg of the shRNA-encoding lentiviral construct (shRNA ID # TRCN0000010677) in the presence of METAFECTENE PRO (Biontex Laboratories, München, Germany). After 16 hours, medium was replaced with regular culture medium. Collected viral particles (after 48 and 72 hours of transfection) were concentrated using virus concentrator Lenti-X concentrator (Clontech Laboratories, Inc. CA, USA) according to the manufacturer’s instructions. Cells were transduced by lentiviral particles in medium containing 10% FBS and 5 μg/mL polybrene (Sigma-Aldrich) in 24-well plates. Cells were incubated for 10–20 minutes at room temperature then plates were centrifuged at 1500 × g for 1 hour and 30 minutes at 35 °C. After centrifugation, cells were incubated at 37 °C and 5% CO_2_ overnight. The lentiviral transduction medium was replaced with a complete culture medium. Cell lines with stable shRNA expression were generated by puromycin selection at 4 μg/mL for 3–4 days. Western blotting was performed to confirm EphB6 silencing.

### Flow cytometry

The indicated T-ALL cells (1 × 10^6^ per well) were incubated with 5 μM doxorubicin for 24 hours. Cells were harvested and washed twice with PBS. Pellets were resuspended in 250 μL 1X binding buffer from PE Annexin V Apoptosis Detection Kit 1 (BD Biosciences) and 100 μL aliquots were transferred to 5 mL flow cytometry tubes. PE-Annexin V reagent was added to each tube and cells were incubated for 15 minutes at room temperature in dark. Cells were resuspended in 1X binding buffer and PE staining was assessed by flow cytometry. Results were analysed using the FlowJo software (FlowJo LLC. Ashland, OR, USA).

### Caspase-3 assay

To analyse caspase-3 activation, cells were seeded in 6-well plates (1 × 10^6^ per well) in FBS-free RPMI-1640 medium. Cells were treated with 5 μM doxorubicin for 14 hours in 37 °C and 5% CO_2_. Cells were harvested, washed once with PBS, lysed and caspase-3 activity was analysed using the EnzCheck Caspase-3 Assay Kit #1 (Invitrogen, Life Technologies, Waltman, MA, USA) according to manufacturer’s instructions. In this assay, caspase-3 activity was quantitated using a SpectraMax M5 microplate reader (Molecular Devices, Sunnyvale, CA, USA).

### Analysis of the synergistic effect of combination treatment with perifosine and doxorubicin

T-ALL cells were treated with a solvent control or with increasing concentrations of doxorubicin alone, or in combination with a suboptimal concentration of perifosine (Invivogen) for 24 hours. Cell survival was monitored by resazurin staining. To assess synergistic effects, the CompuSyn software was used to generate combination indices for each drug combination, as previously described^37^. Indices below 0.9 indicate synergism^37^.

### Xenograft models

Animal protocols were approved by the Animal Research Ethics Board (AREB) at University of Saskatchewan. All *in vivo* experiments were carried out in accordance with relevant guidelines and regulations. Breeder pairs of NOD-SCID mice were from the Jackson Laboratory. ΔB6-Jurkat and pc3-Jurkat were mixed with Corning Matrigel Matrix (Corning, NY, USA) (3 × 10^6^ cells resuspended in 50 μL of PBS were mixed with 50 μL of the matrigel) and injected subcutaneously into the right flanks of experimental animals. Experimental groups injected with pc3-Jurkat or ΔB6-Jurkat cells were of an equal size within each independent experiment (n = 7 or n = 6) and contained equal mixtures of male and female animals. Once tumours reached a measurable size, mice were treated weekly via intravenous injections of doxorubicin at 2 mg/kg or a matching saline control. Tumours were measured using a digital caliper twice a week and tumour volumes were calculated using the following equation: A/2*B^2^, where A was long and B was short diameters of a tumour. All experiments were terminated in accordance with AREB guidelines.

### Statistical analysis

Until otherwise indicated, student’s *t*-test was performed for statistical analyses. Data are presented as the mean ± standard deviation (SD).

### Data availability

Original data files are available upon a reasonable request.

## Electronic supplementary material

Supplementary Information

## References

[1] Belver L, Ferrando A (2016). The genetics and mechanisms of T cell acute lymphoblastic leukaemia. Nat Rev Cancer.

[2] Karrman K, Johansson B (2017). Pediatric T-cell acute lymphoblastic leukemia. Genes Chromosomes Cancer.

[3] Girardi T, Vicente C, Cools J, De Keersmaecker K (2017). The genetics and molecular biology of T-ALL. Blood.

[4] Hebert J, Cayuela JM, Berkeley J, Sigaux F (1994). Candidate tumor-suppressor genes MTS1 (p16INK4A) and MTS2 (p15INK4B) display frequent homozygous deletions in primary cells from T- but not from B-cell lineage acute lymphoblastic leukemias. Blood.

[5] Weng AP (2004). Activating mutations of NOTCH1 in human T cell acute lymphoblastic leukemia. Science.

[6] Bellavia D (2002). Combined expression of pTalpha and Notch3 in T cell leukemia identifies the requirement of preTCR for leukemogenesis. Proc Natl Acad Sci USA.

[7] Bash RO (1995). Does activation of the TAL1 gene occur in a majority of patients with T-cell acute lymphoblastic leukemia? A pediatric oncology group study. Blood.

[8] Ferrando AA (2002). Gene expression signatures define novel oncogenic pathways in T cell acute lymphoblastic leukemia. Cancer Cell.

[9] Gutierrez A (2011). The BCL11B tumor suppressor is mutated across the major molecular subtypes of T-cell acute lymphoblastic leukemia. Blood.

[10] Kees UR (2003). Expression of HOX11 in childhood T-lineage acute lymphoblastic leukaemia can occur in the absence of cytogenetic aberration at 10q24: a study from the Children’s Cancer Group (CCG). Leukemia.

[11] Goldberg JM (2003). Childhood T-cell acute lymphoblastic leukemia: the Dana-Farber Cancer Institute acute lymphoblastic leukemia consortium experience. J Clin Oncol.

[12] Carroll WL (2003). Pediatric acute lymphoblastic leukemia. Hematology Am Soc Hematol Educ Program.

[13] Kania A, Klein R (2016). Mechanisms of ephrin-Eph signalling in development, physiology and disease. Nat Rev Mol Cell Biol.

[14] Lisabeth, E. M., Falivelli, G. & Pasquale, E. B. Eph receptor signaling and ephrins. *Cold Spring Harb Perspect Biol* **5** (2013). 10.1101/cshperspect.a009159 PMC3753714 24003208

[15] Truitt L, Freywald A (2011). Dancing with the dead: Eph receptors and their kinase-null partners. Biochem Cell Biol.

[16] Pasquale EB (2010). Eph receptors and ephrins in cancer: bidirectional signalling and beyond. Nat Rev Cancer.

[17] Himanen JP, Saha N, Nikolov DB (2007). Cell-cell signaling via Eph receptors and ephrins. Curr Opin Cell Biol.

[18] Aasheim HC, Patzke S, Hjorthaug HS, Finne EF (2005). Characterization of a novel Eph receptor tyrosine kinase, EphA10, expressed in testis. Biochim Biophys Acta.

[19] Gurniak CB, Berg LJ (1996). A new member of the Eph family of receptors that lacks protein tyrosine kinase activity. Oncogene.

[20] Matsuoka H (1997). Expression of a kinase-defective Eph-like receptor in the normal human brain. Biochemical & Biophysical Research Communications.

[21] Freywald A, Sharfe N, Roifman CM (2002). The Kinase-null EphB6 Receptor Undergoes Transphosphorylation in a Complex with EphB1. J Biol Chem.

[22] Truitt L, Freywald T, DeCoteau J, Sharfe N, Freywald A (2010). The EphB6 receptor cooperates with c-Cbl to regulate the behavior of breast cancer cells. Cancer Res.

[23] Matsuoka H, Obama H, Kelly ML, Matsui T, Nakamoto M (2005). Biphasic functions of the kinase-defective Ephb6 receptor in cell adhesion and migration. J Biol Chem.

[24] Freywald A, Sharfe N, Rashotte C, Grunberger T, Roifman CM (2003). The EphB6 receptor inhibits JNK activation in T lymphocytes and modulates T cell receptor-mediated responses. J Biol Chem.

[25] Luo H, Yu G, Tremblay J, Wu J (2004). EphB6-null mutation results in compromised T cell function. J Clin Invest.

[26] Lu P, Shih C, Qi H (2017). Ephrin B1-mediated repulsion and signaling control germinal center T cell territoriality and function. Science.

[27] Maddigan A (2011). EphB receptors trigger Akt activation and suppress Fas receptor-induced apoptosis in malignant T lymphocytes. J Immunol.

[28] Asselin BL (2016). Cardioprotection and Safety of Dexrazoxane in Patients Treated for Newly Diagnosed T-Cell Acute Lymphoblastic Leukemia or Advanced-Stage Lymphoblastic Non-Hodgkin Lymphoma: A Report of the Children’s Oncology Group Randomized Trial Pediatric Oncology Group 9404. J Clin Oncol.

[29] Shimoyama M (2000). T-cell-specific expression of kinase-defective Eph-family receptor protein, EphB6 in normal as well as transformed hematopoietic cells. Growth Factors.

[30] Julien O, Wells JA (2017). Caspases and their substrates. Cell Death Differ.

[31] Soldani C, Scovassi AI (2002). Poly(ADP-ribose) polymerase-1 cleavage during apoptosis: an update. Apoptosis.

[32] Mora A, Komander D, van Aalten DM, Alessi DR (2004). PDK1, the master regulator of AGC kinase signal transduction. Semin Cell Dev Biol.

[33] Teixeiro E, Daniels MA (2010). ERK and cell death: ERK location and T cell selection. FEBS J.

[34] Masiero M (2011). Notch3-mediated regulation of MKP-1 levels promotes survival of T acute lymphoblastic leukemia cells. Leukemia.

[35] Indraccolo S (2006). Interruption of tumor dormancy by a transient angiogenic burst within the tumor microenvironment. Proc Natl Acad Sci USA.

[36] Roti G, Stegmaier K (2014). New Approaches to Target T-ALL. Front Oncol.

[37] Chou TC, Talalay P (1984). Quantitative analysis of dose-effect relationships: the combined effects of multiple drugs or enzyme inhibitors. Adv Enzyme Regul.

