# Supplementary material for: The EphB6 receptor is overexpressed in pediatric T cell acute lymphoblastic leukemia and increases its sensitivity to doxorubicin treatment
Source: Sci Rep. 2017 Nov 7;7:14767. doi: 10.1038/s41598-017-15200-3 (PMC5676711; doi:10.1038/s41598-017-15200-3)
Supplement: Supplementary file 1 — Supplementary Information [file 41598_2017_15200_MOESM1_ESM.pdf]

The EphB6 receptor is overexpressed in pediatric T cell acute lymphoblastic leukemia and increases its sensitivity to doxorubicin treatment

Amr El Zawily, Emily McEwen, Behzad Toosi, Frederick S. Vizeacoumar, Tanya Freywald, Franco J. Vizeacoumar, and Andrew Freywald

Supplementary Figure Legends

Supplementary Figure 1

A. Full- length unadjusted images of Western blots shown in Figure 2A
B. Full- length unadjusted images of Western blots shown in Figure 2C
C. Full- length unadjusted images of Western blots shown in Figure 2E

Supplementary Figure 2

A. Full- length unadjusted images of Western blots shown in Figure 3C

Supplementary Figure 3

A. Full- length unadjusted images of Western blots shown in Figure 4A
B. Full- length unadjusted images of Western blots shown in Figure 4B
C. Full- length unadjusted images of Western blots shown in Figure 4C

A

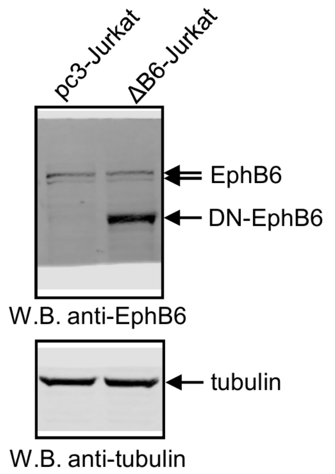
pc3-Jurkat
ΔB6-Jurkat
EphB6
DN-EphB6
W.B. anti-EphB6
tubulin
W.B. anti-tubulin

B

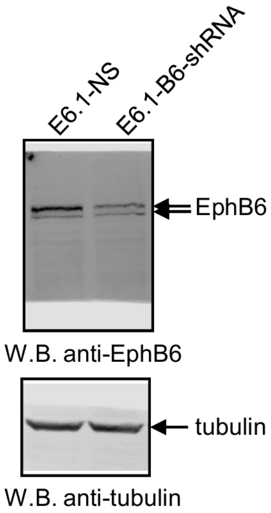
E6.1-NS
E6.1-B6-shRNA
EphB6
W.B. anti-EphB6
tubulin
W.B. anti-tubulin

C

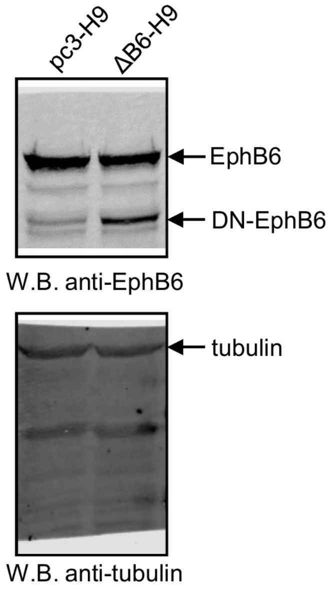
pc3-H9
ΔB6-H9
EphB6
DN-EphB6
W.B. anti-EphB6
tubulin
W.B. anti-tubulin

A

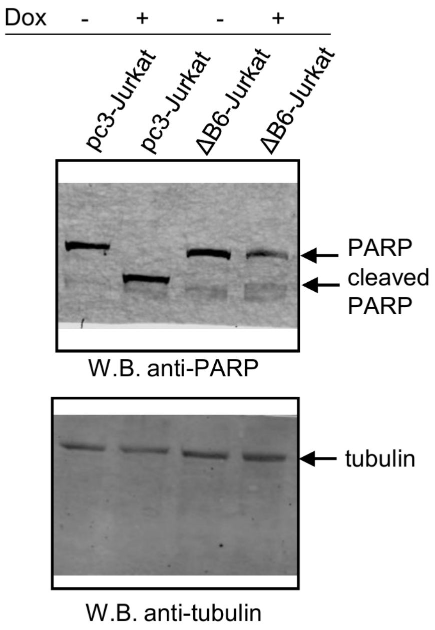
Dox - + - +
pc3-Jurkat
pc3-Jurkat
ΔB6-Jurkat
ΔB6-Jurkat
PARP
cleaved PARP
W.B. anti-PARP
tubulin
W.B. anti-tubulin

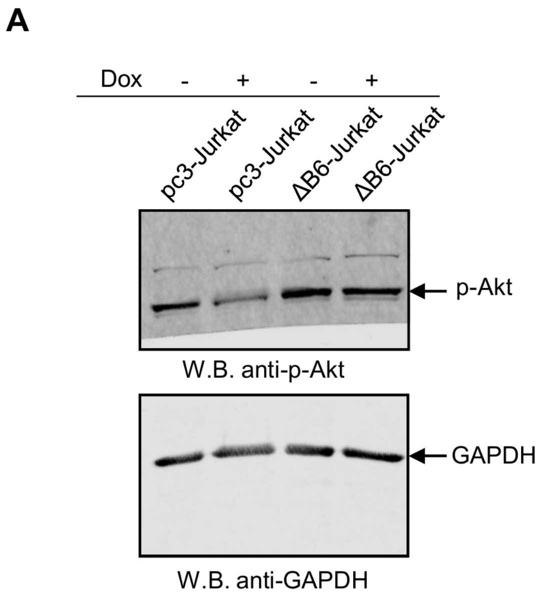
A
Dox - + - +
pc3-Jurkat
pc3-Jurkat
ΔB6-Jurkat
ΔB6-Jurkat
p-Akt
W.B. anti-p-Akt
GAPDH
W.B. anti-GAPDH

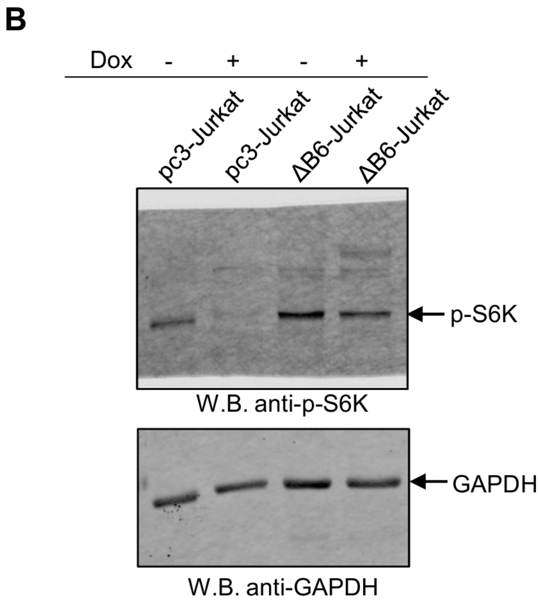
B
Dox - + - +
pc3-Jurkat
pc3-Jurkat
ΔB6-Jurkat
ΔB6-Jurkat
p-S6K
W.B. anti-p-S6K
GAPDH
W.B. anti-GAPDH

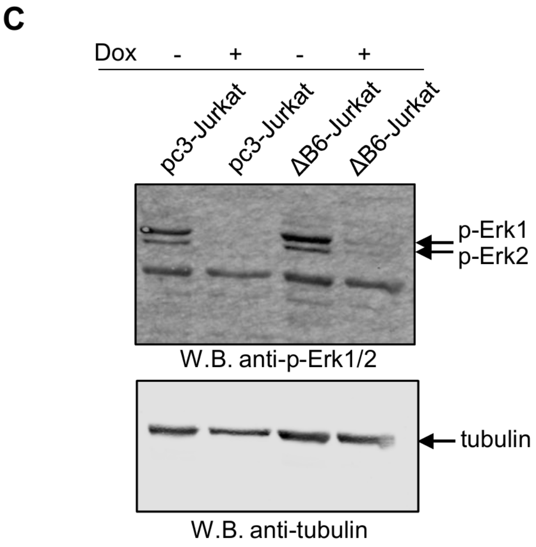
C
Dox - + - +
pc3-Jurkat
pc3-Jurkat
ΔB6-Jurkat
ΔB6-Jurkat
p-Erk1
p-Erk2
W.B. anti-p-Erk1/2
tubulin
W.B. anti-tubulin
